# Supplementary material for: Comprehensive Annotation and Functional Exploration of MicroRNAs in Lettuce
Source: Front Plant Sci. 2021 Dec 24;12:781836. doi: 10.3389/fpls.2021.781836 (PMC8739914; doi:10.3389/fpls.2021.781836)
Supplement: Supplementary file 3 [file Data_Sheet_9.ZIP › GSM2230753.plot/Lsa-miR6114_Lsat_1_v5_gn_8_23661.1_184_TPlot.pdf]

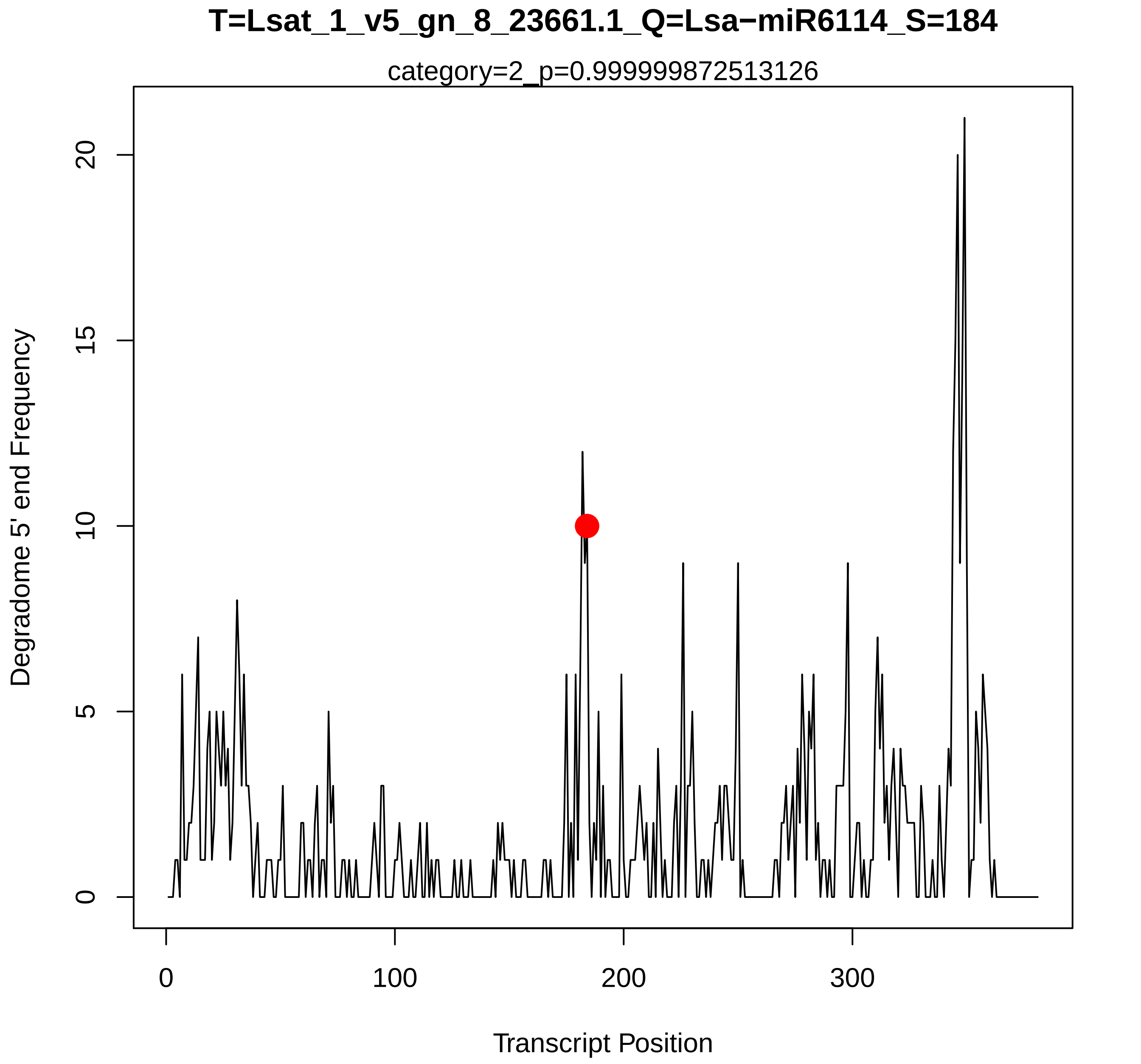

T=Lsat_1_v5_gn_8_23661.1_Q=Lsa−miR6114_S=184
category=2_p=0.999999872513126
Degradome 5' end Frequency
0
5
10
15
20
0
100
200
300
Transcript Position
